# Human Papillomavirus Oral- and Sero- Positivity in Fanconi Anemia

**DOI:** 10.3390/cancers13061368

**Published:** 2021-03-18

**Authors:** Sharon L. Sauter, Xue Zhang, Lindsey Romick-Rosendale, Susanne I. Wells, Kasiani C. Myers, Marion G. Brusadelli, Charles B. Poff, Darron R. Brown, Gitika Panicker, Elizabeth R. Unger, Parinda A. Mehta, Jack Bleesing, Stella M. Davies, Melinda Butsch Kovacic

**Affiliations:** 1 Cincinnati Children’s Hospital Medical Center, Department of Pediatrics, University of Cincinnati College of Medicine, Cincinnati, OH 45229, USA; sharon.sauter@cchmc.org (S.L.S.); xue.zhang@cchmc.org (X.Z.); lindsey.romick-rosendale@cchmc.org (L.R.-R.); susanne.wells@cchmc.org (S.I.W.); kasiani.myers@cchmc.org (K.C.M.); M.Brusadelli@medpace.com (M.G.B.); poffc@musc.edu (C.B.P.); parinda.mehta@cchmc.org (P.A.M.); jack.bleesing@cchmc.org (J.B.); stella.davies@cchmc.org (S.M.D.); 2 Division of Infectious Diseases, Indiana University School of Medicine, Indianapolis, IN 46202, USA; darbrow@iu.edu; 3 National Center for Emerging and Zoonotic Infectious Diseases, Centers for Disease Control and Prevention, Atlanta, GA 30329, USA; dhv1@cdc.gov (G.P.); eru0@cdc.gov (E.R.U.); 4 Department of Rehabilitation, Exercise and Nutrition Sciences, University of Cincinnati College of Allied Health Sciences, Cincinnati, OH 45267, USA

**Keywords:** head and neck squamous cell carcinoma, oropharyngeal cancer, human papillomavirus, Fanconi anemia, oral HPV positivity, seropositivity, HPV vaccine

## Abstract

**Simple Summary:**

People with Fanconi anemia (FA) are genetically susceptible to gynecological cancers and cancers of the head and neck. There are known associations between oral infection with human papillomavirus (HPV) and development of head and neck cancers. This study sought to measure how common oral HPV positivity is in a large sample of people with FA followed over 8 years, while also evaluating serum titers to ascertain natural exposure to HPV, and how well people with FA who were vaccinated responded to HPV vaccination. We found that oral HPV positivity is significantly higher in individuals with FA compared to family and unrelated controls, but that response to HPV vaccination between FA and controls is similar. Common risk factors associated with HPV in the general population did not predict oral DNA positivity in FA, unlike unrelated controls. Future mechanistic and vaccinations studies are needed to understand this phenomenon.

**Abstract:**

High-risk human papillomavirus (HPV) is prevalent and known to cause 5% of all cancers worldwide. The rare, cancer prone Fanconi anemia (FA) population is characterized by a predisposition to both head and neck squamous cell carcinomas and gynecological cancers, but the role of HPV in these cancers remains unclear. Prompted by a patient-family advocacy organization, oral HPV and HPV serological studies were simultaneously undertaken. Oral DNA samples from 201 individuals with FA, 303 unaffected family members, and 107 unrelated controls were tested for 37 HPV types. Serum samples from 115 individuals with FA and 55 unrelated controls were tested for antibodies against 9 HPV types. Oral HPV prevalence was higher for individuals with FA (20%) versus their parents (13%; *p* = 0.07), siblings (8%, *p* = 0.01), and unrelated controls (6%, *p* ≤ 0.001). A FA diagnosis increased HPV positivity 4.84-fold (95% CI: 1.96–11.93) in adjusted models compared to unrelated controls. Common risk factors associated with HPV in the general population did not predict oral positivity in FA, unlike unrelated controls. Seropositivity and anti-HPV titers did not significantly differ in FA versus unrelated controls regardless of HPV vaccination status. We conclude that individuals with FA are uniquely susceptible to oral HPV independent of conventional risk factors.

## 1. Introduction

High-risk human papillomavirus (HPV) is prevalent and is known to cause 5% of all cancers worldwide [1,2]. According to the Centers for Disease Control, 14 million sexually active Americans, frequently teens and young adults, are newly infected with HPV each year [3]. High-risk HPV types, particularly HPV16, are associated with gynecological, anogenital and oropharyngeal squamous cell carcinoma in the general population, and transmission is thought to occur mainly via sexual contact including oral, vaginal, and anal sex, and open-mouthed kissing [4,5,6]. Other routes of transmission, such as fomites and fingers, are also possible [7,8]. While 90% of infections resolve within two years (no detection by PCR), factors associated with viral persistence, the major risk factor for cancer development, include, viral load, oncogene expression and compromised immunity [9].

Some populations are at greater risk of HPV-associated cancer than others [10]. Notably, individuals with Fanconi anemia (FA) have more than a 1000-fold greater risk of head and neck squamous cell carcinoma (HNSCC) [11], and increased prevalence of oral HPV [12]. FA is a rare (estimated 31 babies born in the US/year) inherited bone marrow failure disorder characterized by defects in one of at least 22 FA or FA-like genes in the FA DNA repair pathway [13,14]. Those affected are at greater risk of HNSCC and anogenital cancers that are often associated with HPV, as well as myelodysplastic syndrome and leukemia—all with dismal survival rates [15,16]. FA is often diagnosed years before the onset of severe bone marrow failure or cancer, and cancer risk in FA does not diminish, and may actually increase following treatment of bone marrow failure by hematopoietic stem cell transplant (HSCT) [17]. This makes FA an ideal model population to longitudinally identify modifiable risk factors for cancer prevention and better understand sporadic cancers with similar genetic defects [18,19,20].

We have previously reported that loss of function of the FA-related DNA repair pathway in high-risk HPV positive epidermis stimulates production of the E7 oncogene, increases viral replicative capacity, and expands the basal stem and progenitor cell compartment [9,21]. These data show that the FA pathway is linked to HPV biology and epidermal pathogenesis, and the FA population represents a model for HPV vulnerabilities.

Our earlier epidemiologic work in this area was first prompted by the Fanconi Anemia Research Fund (FARF), a patient-family advocacy organization with a governing board of patients, family members, clinicians and scientists. In response to their call, we sought to understand the nature of HPV susceptibility in FA by characterizing the epidemiology of oral HPV infections and host immunological responses in individuals with FA and family controls, considering that all parents of those with FA, and a portion of siblings are heterozygotes with mutations in one of the 22 Fanconi genes. We found that participants with FA are particularly susceptible to oral HPV infection with 11.1% of FA individuals testing oral HPV DNA positive to at least one of 37 HPV types by Roche Linear Array, significantly more than first degree family members (2.5%; *p* = 0.003). Interestingly, even those with FA who had not been sexually active tested oral HPV DNA positive more often than siblings, although the difference was not statistically significant (8.7% vs. 2.9% *p* = 0.44) [12]. Increased viral susceptibility, potentially due to decreased immune function, could explain the higher oral HPV prevalence in participants with FA [22]. However, based on the rare nature of this unique cancer prone population, longitudinal insights have been limited.

Over a period of eight years, we consented one of the largest cohorts of families to date, to test oral HPV DNA prevalence in individuals with FA compared to siblings, parents and unrelated controls, as well as take an early look at the incidence and persistence of oral HPV positivity. This allowed for close examination of risk factors typically associated with HPV infection in the general population including early life exposures, hygiene practices, use and exposure to tobacco products, alcohol consumption and sexual practices. Further, antibody responses to HPV are generally type-specific making evaluation of antibodies in blood serum one of the best approaches to simultaneously monitor response to natural infection and gauge the protective effect of HPV vaccines [23]. Consequently, we concomitantly assessed HPV seropositivity in a subset of unvaccinated and vaccinated individuals with FA compared to unrelated controls, considering transplant history. The resulting body of data supports a surprising scenario wherein individuals with FA harbor a unique susceptibility for oral HPV positivity that is independent of conventional risk factors.

## 2. Materials and Methods

### 2.1. Participant Recruitment

At the invitation of the Fanconi Anemia Research Fund (FARF), we invited attendees of the FARF’s Adult and Family Meetings to participate. Additionally, patients visiting the Cincinnati Children’s FA Comprehensive Care Center were also invited. Participants were from 15 countries and 34 states across the USA. Individuals of all ages were eligible to participate if they reported a diagnosis of FA or were a parent or sibling of an individual with FA and were willing to complete study-related surveys and provide oral rinse samples. Healthy controls were recruited either at Cincinnati Children’s Hospital Medical Center or at a FARF sponsored event and were eligible if they did not report a diagnosis of FA, did not have a family member with FA, and had no other known bone marrow or immune abnormalities. A total of 212 subjects with FA were enrolled in the study, as well as 331 family members (biological siblings and parents), and 111 unrelated controls. Of these, 201 individuals with FA, 88 siblings, 215 parents and 107 unrelated controls have at least one sample for HPV DNA testing, while 128 participants with FA and 151 family members have two or more samples tested over an 8-year period. In addition, 115 individuals with FA and 55 unrelated controls had at least one blood sample for antibody testing. The majority of control samples were collected at a single time point at various times throughout the life of the study. The Institutional Review Board at Cincinnati Children’s Hospital Medical Center approved this study.

### 2.2. Risk Factor Survey

The surveys were typically self-administered either via paper (years 1 and 2 only) or online (Research electronic data capture (REDCap) software) [24]. Participants ages 15 years and older were asked to complete surveys for themselves unless there was a developmental delay, in which case a parent or guardian was asked to complete the survey. For children ages 12 to 15 years, a parent was asked to complete the survey or assist the child in completing the survey. However, answers to the sexual questions were ascertained by study staff interview whenever possible. Surveys collected demographic and clinical data including FA complementation group, history of cancer, disease severity, and date/age at hematopoietic stem cell transplant (if applicable). In addition, other potential risk factors for HPV-related disease such as vaginal versus cesarean section mode of delivery at birth, gestational age at birth, tobacco smoke exposure, alcohol consumption and sexual history (including questions about oral sex and kissing), family history of FA and cancer, history of personal immunization and/or HPV testing, and presence and frequency of oral lesions were ascertained by survey and/or staff interview. Second-hand tobacco smoke exposure was defined by indicating regular exposure to others who smoke cigarettes or cigars in their presence. Survey responses for demographics (Table 1) was taken from the first non-missing time point. The most recent non-missing time point was used for all other tables.

### 2.3. Oral Sample Collection and HPV DNA Testing

Oral rinse and gargle samples were collected in 15 mL normal sterile saline and processed as previously described [12]. Briefly, DNA was isolated using the 5 Prime ArchivePure DNA kit (5 Prime Inc., Gaithersburg, MD, USA) adhering to the manufacturers protocol. Beta globin is a housekeeping gene that was included to verify that the PCR conditions were optimal and that the samples contained adequate levels of DNA. The Linear Array HPV Genotyping Test (Roche Molecular Diagnostics, Indianapolis, IN, USA) [25] was used to detect 37 HPV types including types 6, 11, 16, 18, 26, 31, 33, 35, 39, 40, 42, 45, 51, 52, 53, 54, 55, 56, 58, 59, 61, 62, 64, 66, 67, 68, 69, 70, 71, 72, 73, 81, 82, 83, 84, IS39, and CP6108 (HPV89), as previously described. Given our study’s collaboration with the FARF, oral HPV test results and educational information about HPV were shared with participants with FA in the form of a letter.

### 2.4. Serum Sample Collection, Processing and HPV Antibody Testing

Blood samples were collected using appropriate precautions by trained personnel. Fifty percent of the samples from participants with FA were collected at Cincinnati Children’s Hospital, with the remainder collected at FARF events as previously described [26]. Eighty-three percent of healthy control samples were collected at Cincinnati Children’s Hospital with the remainder collected from spouses and friends who attended FARF events with those with FA. Serum samples were sent by priority mail to the Centers for Disease Control and Prevention (CDC) in two batches. For the first batch, HPV antibody testing was performed targeting antibodies contained in the 4-valent Gardasil vaccine using a direct IgG enzyme-linked immunosorbent assay ELISA (M4ELISA) on Meso Scale Discovery platform (MSD, Gaithersburg, MD) using HPV L1 + L2 virus-like particles (VLPs) for HPV6, 11, 16, 18 at the CDC (Atlanta, GA, USA) as previously described with minor modifications [27]. For the second batch, the M9ELISA was done targeting the 9-valent Gardasil vaccine. The M9ELISA used VLPs for HPV6, 11, 16, 18, 31, 33, 45, 52 and 58 pre-coated on 10-spot standard plates (MSD). Test sera were 3.16-fold serially-diluted for at least 3 dilutions starting at 1:100 or higher. Dilutions of reference sera were used in each plate to allow titer determination using the parallel line method (PLL). PLL analysis was performed as described in the WHO HPV Labnet Manual, using raw signal for each HPV type. Test samples were considered seropositive if they passed PLL conditions as well as were above Median+2 Standard Deviations of the PLL/titer generated from the children’s sera (Gift from Dr. J Dillner, Lund University). Cut off values for HPV6, 11, 16, 18, 31, 33, 45, 52 and 58 were 0.3 AU/mL, 0.3 AU/mL, 0.7 IU/mL, 1.2 IU/mL, 1.8 AU/mL, 2.6 AU/mL, 3.4 AU/mL, 1.5 AU/mL and 3.0 AU/mL, respectively. M9ELISA results were used if available, unless a participant only had a M4ELISA result.

### 2.5. Statistical Analysis

Statistical analyses were performed using Statistical Analysis Software (SAS), version 9.4 (SAS Institute Inc., Cary, NC, USA). Prior to analyses, data qualities were examined. Demographic variables and potential risk factors of HPV were summarized as frequencies (proportions) and compared between FA, siblings, parents and unrelated controls using chi-square or Fisher’s exact tests. These factors were then compared between oral HPV negative and positive individuals with FA, and in unrelated controls. The effects of HPV risk factors on HPV positivity were also tested using multi-variable logistic regression.

For our analysis of oral HPV prevalence, any sample testing positive with any HPV type was considered oral HPV positive. For our analysis of risk, high-risk types 16, 18, 35, 45, 51, 52, 56, 58, 59, 66, 68, were included in our high-risk group; all others were considered low risk. For the analysis, if a sample tested positive for a high-risk type (regardless of having low risk types), they were in the high-risk category; the low-risk category only included participants with low risk types. A similar analysis was performed focusing only on those types targeted in the 4-valent Gardasil vaccine. Multiple HPV positive results over events includes participants testing positive for multiple HPV types in a single sample. Cumulative incidence was defined as the proportion of new HPV positive cases during the entire study over the total subjects at risk (subjects that tested negative when entering the study). Participants with no oral sample (37), those with samples inadequate for evaluation (negative for beta globin, 11) and those positive for HPV in the first/baseline test (48) were removed from our analysis of incidence. Of the remaining 558 subjects, 114 individuals with FA, 105 parents, 35 siblings, and 6 unrelated controls had ≥2 oral test results. Those with more than one sample had up to 6 follow-up time points with median time between sampling events being 1.13 years. Because only 6 unrelated controls had testing at more than one-time point, they were not included in our analysis of incidence. Finally, consecutive oral HPV positivity was defined as participants testing positive for any HPV type in subsequent samples (having the same HPV type across samples). Serotiters were analyzed using Wilcoxon rank sum tests. Titers under the detection limits were imputed as half of the detection limits. Geometric mean titers (GMT) were calculated and compared between groups using a student t-test assuming log normal distribution. Statistical significance was indicated by a *p*-value ≤ 0.05.

## 3. Results

### 3.1. Population Characteristics

A total of 654 individuals with oral rinse and gargle samples and/or risk factor surveys were included in this study. Participants came from 15 countries and 34 states across the USA. Of these, 212 individuals had been diagnosed with FA, 98 were siblings of the individuals with FA, 233 were parents of individuals with FA, and 111 were unrelated controls (Table 1). Age differed between groups even when parents were excluded (*p* < 0.001) (Table 1). Among individuals with FA, 44% were children less than or equal to 11 years of age, and nearly 29% were adults aged 21 and older. Siblings were overall younger than those with FA and unrelated controls, with 60% being less than or equal to 11 years of age and only 8% being 21 years or older. Only 29% of unrelated controls were less than or equal to 11 years of age and 37% were 21 or older. Though gender and race did not statistically differ by groups, there were more females in each group than males, and more Caucasians than any other race. Individuals with FA were living in families across all income levels, and most had some type of health insurance coverage. Unrelated controls were the most likely to have private insurance, 81%, versus 56% of individuals with FA (*p* < 0.001). Overall, the parents of those with FA were highly educated; only 16% and 23% of fathers and mothers, respectively, having less than a college education (Table 1). More than 70% of adults with FA were college educated or seeking post-graduate education.

### 3.2. Oral HPV Positivity

Oral HPV positivity was evaluated among all study participants with oral samples regardless of their HPV vaccination status. Among individuals with FA, 41 (20%) tested oral positive for at least one type of HPV at one or more time points. The oral HPV prevalence in FA was significantly higher than in unrelated controls (6%), (*p* < 0.001); it was also higher than their first-degree family members, parents (13%, *p* = 0.07) and siblings (8%, *p* = 0.010) respectively (Fisher’s exact test) (Table 2). Considering all participants, 90% tested positive for at least one high-risk HPV type, and 80% were oral positive for one or more of the HPV types (HPV6, 11, 16, 18) targeted in the 4-valent Gardasil vaccine (Table 2). The prevalence of HPV6, 11, 16, and 18 did not significantly differ by vaccination status across the subject groups (*p* = 0.90) or within the FA group (*p* = 0.77). Indeed, among 18 oral DNA positive individuals with FA who had not been vaccinated for HPV at the time of sampling, 14 (78%) were oral HPV positive for a type targeted in the 4-valent vaccine. Among the 23 (56%) oral positive vaccinated individuals with FA, 19 (83%) tested oral HPV DNA positive for a type targeted in the 4-valent vaccine. There were only three vaccinated siblings and four unvaccinated siblings testing positive to a 4-valent type. No parents or unrelated controls who tested oral positive had been vaccinated at the time of sampling.

The most common HPV type detected across groups was HPV16, consistent with published studies [28,29]. Indeed, 76% of the individuals with FA, all but one sibling, and approximately half of parents and controls tested positive for HPV16. The next most common types of HPV detected among individuals with FA were HPV6 (15%) and HPV18 (13%). Other HPV types detected in oral samples from those with FA include HPV42, 51, 58, 59, 61, 62, 66, 84 and CP6108 (HPV89). Among all control groups, HPV35, 45, 51, 52, 56, 58, 62, 64, 66, 68 and 84 were also detected.

When stratified by age, those 12 years and older with FA were significantly more likely to test oral HPV positive (22%) than siblings (7%, *p* = 0.025), parents (12%, *p* = 0.018), and unrelated controls (7%, *p* = 0.004). In participants less than 12 years old, oral HPV positivity did not significantly differ in those with FA (15%) compared to siblings (10%, *p* = 0.55) or unrelated controls (3%, *p* = 0.16). Notably, in oral HPV positive individuals with FA, 13 (32%) had multiple HPV types in the same sample, including two individuals younger than 12 years old. None of the oral HPV positive siblings, 3 parents (10%), and only a single unrelated control (17%) had multiple oral HPV types detected (Table 2).

There were 21 new HPV positive samples identified among individuals with FA during the study period, with a cumulative incidence of 18%. In parents and siblings, 9 and 3 new HPV positive samples were observed, with cumulative incidences of 9% and 9%, respectively. The cumulative incidence in FA was significantly higher when compared with parents (*p* = 0.048), but did not reach statistical significance when compared with siblings (*p* = 0.20) (Table 2). Given the nature of the cohort’s recruitment at FARF meetings and at visits to Cincinnati Children’s Hospital Medical Center and often their younger ages, siblings were not always available to provide samples for testing, reducing the overall sample size of this important group.

Looking over time, only five individuals with FA tested oral HPV positive in consecutive samplings compared to four parents (Table 2). Type-specific persistence was even more rare. Only three of the individuals with FA (HPV51 and HPV84 and HPV42) and two fathers (HPV51 and HPV6) tested positive for the same HPV types in consecutive samplings collected within 1.5 years of each other (Table 2). Of interest, a participating female patient with FA in her early 20’s at Cincinnati Children’s Hospital Medical Center was diagnosed with oral and vulvar squamous cell carcinoma. While we were unable to confirm that her tumor samples were HPV positive, over four study time points (over a 2.5-year time period), she persistently tested positive in oral rinses for HPV84. She also tested positive for HPV42 at two time points, and HPV61 and HPV16 at one-time point each. Examining family units, one father had a persistent oral HPV51 infection that was maintained through four events. His child with FA tested positive for HPV16 in oral rinse at one-time point, and was HPV negative thereafter.

### 3.3. Exposure to HPV

We next assessed exposure and behavior-related factors that could influence positivity between case and control groups (Table 3). Delivery varied across the groups with 90% of parents being delivered vaginally versus 71% of individuals with FA (*p* < 0.001) (Table 3). In comparison to the sibling and unrelated control groups, the FA group did not differ for delivery and having been breast fed as an infant. However, individuals with FA were significantly more likely to have been born pre-term (28% vs. 15% *p* = 0.03 and 10% *p* < 0.001 respectively) and at lower birth weight (35% vs. 4% and 10% *p* < 0.001 respectively) (Table 3).

With regard to oral hygiene, the siblings’ brushing and flossing habits were more similar to the FA group; but significant differences were observed between FA and parents and unrelated controls (Table 3). Not surprisingly, given the clinical recommendations [30], those with FA were more likely to have three or more dental visits per year (13%, vs. 4% and 5% for the other groups). Importantly, individuals with FA reported significantly more mouth sores (30%) compared to all other groups (*p* < 0.001). This difference was not influenced by a history of HSCT.

Given the considerable age differences, exposures later in life including alcohol use and current and ever smoking were significantly higher for parents compared to those with FA, siblings and unrelated controls (*p* < 0.001, respectively). However, exposure to second-hand smoke did not significantly differ between groups (*p* = 0.29; Table 3). Forty-five percent of individuals with FA reported receiving the HPV vaccine (primarily 4-valent Gardasil vaccine) compared to siblings (24%) and unrelated controls (34%); only 3 parents reported receiving the vaccine (1%) (all control *p* = 0.002; Table 3). Thirty-four percent of individuals with FA reported ever having a sexual experience compared to 20% of siblings and 48% of unrelated controls (Table 3). Among those who have had sexual experience, there were no significant differences between groups in specific sexual behaviors, except that all parents reported ever having had vaginal sex (*p* = 0.014) (Table S1).

As some sexual behaviors may lead to increased risk of sexually transmitted diseases (STDs), we next examined differences between groups in reported STDs (Table 4). Those with FA who were sexually active reported significantly more genital HPV and chlamydia compared to parents and unrelated controls (*p* < 0.003 and *p* = 0.05). Indeed, 31% of individuals with FA reported ever having had a STD vs. 12% of siblings, 14% of parents and 13% of controls (all control *p* = 0.012) (Table 4). Sexual behaviors were similar to controls although with later onset.

Among unrelated controls, only 6 tested positive for oral HPV; 2 males and 4 females. None of the risk factors examined were significantly associated with oral HPV positivity. However, with the exception of sex (male: female OR = 0.53), the associated trends in our unrelated controls were similar to those previously reported [5,29], with alcohol use increasing odds of oral positivity by 4.31, sexual history increasing odds of oral positivity by 3.75, and second hand smoke (SHS) increasing the odds of positivity to 2.41. Surprisingly, similar trends were not observed in those with FA (Figure S1).

Among subjects with FA, only sex and race were significantly associated with oral HPV positivity (Table 5). Males had 2.34 greater odds of a positive test than females (OR = 2.34; 95% CI: 1.16–4.73), similar to reported studies of non-FA populations [5,29]. Non-white participants had reduced odds of oral HPV positivity compared to whites (OR = 0.11; 95% CI: 0.01–0.82). While some studies have shown that HSCT might be associated with the increased risk of HNSCC or other SCC in individuals with FA [11,31], we found that HPV positivity was not significantly different between the transplanted and not-transplanted groups (OR = 0.88; 95% CI: 1.96–11.93) (Table 5). None of the other potential risk factors examined, such as age at diagnosis of FA, FA complementation group, or personal or family history of cancer, were associated with oral HPV positivity. Indeed, while 43 individuals with FA reported having ever had cancer, only 8 participants tested positive during our study; therefore, ever having cancer was not significantly associated with oral HPV positivity (Table 5). Oral HPV positivity was marginally positively associated with gynecological cancers in females with FA (*p* = 0.08), but not head and neck and oral cancers (*p* = 0.36). At least 46 participants (21.7%) with FA have died (all causes) since the cohort was first initiated.

Considering only those with FA and unrelated controls, multivariable models including FA status, gender and race indicated that having FA was the greatest risk factor (OR = 4.84; 95% CI: 1.96–11.93) for being oral HPV positive.

### 3.4. HPV Seropositivity

HPV seropositivity indicates prior exposure to HPV in unvaccinated individuals and level of response to HPV vaccination in those who have been vaccinated. We therefore focused our serology testing on types targeted in the 9-valent Gardasil vaccine (which include the four types in the 4-valent vaccine). Among unvaccinated individuals, differences in seropositivity between individuals with FA and unrelated controls were not statistically significant (Table 6). Among those vaccinated, only HPV58 was marginally different; those with FA had 25% lower seropositivity than unrelated controls (*p* = 0.05) (Table 6). Because transplantation could alter seropositivity [17], we also compared individuals with FA who had not been transplanted to unrelated controls, also without a history of transplant. We observed a pattern of low (marginally positive) GMT’s in both the unvaccinated, non-transplanted individuals with FA and unvaccinated, unrelated controls; however, responses to individual types were not significantly different (Table S2). Although anti-HPV titers for most types were higher in the vaccinated, unrelated controls compared to the vaccinated, non-transplanted FA group, we observed no significant differences in GMTs (Table S2).

We next compared anti-HPV titers in non-transplanted individuals with FA to those transplanted, stratified by vaccination history. Only post-transplant vaccinations were included in the analysis. There were no statistically significant differences between the median time of sampling for this study and time post HPV vaccination for non-transplanted (median = 3 years; range 2–6 years) and transplanted groups (median 3 years; range 1-6 years) (*p* = 0.27). Non-transplanted participants with FA had higher GMTs than the transplanted FA group regardless of vaccination status consistent with our previous findings [26] (Table 7). Significant differences were observed with HPV18, 31 and 58. (Table 7). For all three HPV types, GMTs in the vaccinated, non-transplanted FA group were approximately three times higher than in the vaccinated, transplanted FA group (*p* = 0.029, 0.05 and 0.015). Notably, HPV31 and 58 are not represented in the original 4-valent Gardasil vaccine, but have been added to the 9-valent vaccine.

As the HPV vaccine is most protective prior to onset of sexual activity, we also examined mean titers of unvaccinated children who were 13 years and younger at sampling and who reported no prior sexual history (Table S3). We found 30 of 46 children with FA (65%) and 11 of 19 unrelated controls (58%) tested positive to one or more HPV types (Table S3). We observed a wide range of titers in both groups, indicating that early exposure to HPV is common across groups. Among seropositive children, seven participants with FA (23%) and one control (5%) ever had oral HPV detected during the study. While the odds of oral HPV DNA detection were higher in individuals with FA compared to unrelated controls, these differences were not statistically significant (OR = 3.04; 95% CI 0.33–28.1) (Table S3).

## 4. Discussion

Previously, we reported that oral HPV prevalence is significantly higher in individuals with FA compared to family members [12]. The current larger study confirms our previous findings and further indicates that detection of oral HPV is also significantly higher in the 201 individuals with FA versus the 107 unrelated controls. This is interesting given that individuals with FA generally have greater rates of HPV vaccination while more unrelated controls reported histories of sexual activity before sampling (Table 2 and Table 3). The incidence of oral HPV DNA detection was overall low; but significantly greater in individuals with FA compared to their parents (*n* = 215) who all are obligate carriers of a single mutation in one of the FA genes (Table 2). Type-specific persistence of oral HPV, too, was low and did not significantly differ between individuals with FA and either the family or unrelated control groups (Table 2).

Among unrelated controls, factors increasing the odds of oral HPV detection were consistent with published studies [5,6,29] (Figure S1). In stark contrast, for individuals with FA, odds ratios were considerably lower than unrelated controls. Further, there were no differences in oral HPV positivity by enrollment age, transplant status or previous sexual experience (Table 5). Only sex and race significantly predicted oral HPV detection with males having greater risk than females (OR = 2.34; CI 0.21–0.86; Table 5) consistent with studies of oral HPV prevalence in the general population [5,29] and studies of HNSCC [32]. Taken together, our data suggests that individuals with FA may have a unique susceptibility to oral HPV positivity that is independent of conventional risk factors, unlike what was observed in the unrelated controls.

We also evaluated many potential risk factors previously associated with either oral DNA HPV positivity or FA to better understand susceptibility to oral HPV in individuals with FA. Sexual contact is the primary mode of transmission for the general population [33]. Acknowledging the general limitations associated with self-reported sexual histories, in this study, among sexually active participants, sexual practices across FA and control groups were similar (Table S1). However, fewer individuals with FA ever reported having had sex compared to similarly aged unrelated controls suggesting that sexual contact alone does not explain the observed higher prevalence of oral HPV in individuals with FA (Table 3 and Table S1). In addition, among those sexually active, individuals with FA reported ever having had a STD twice as often as all other control groups, with chlamydia and genital HPV being the most commonly reported STD (Table 4), suggesting a faulty immune response might contribute to susceptibility.

We previously reported that children and adults with FA had heterogeneous immune defects (both cellular and humoral) that may play a role in viral susceptibility [22,34]. In the current study, we demonstrate that participants with FA are similarly exposed to HPV in childhood (considering childhood serotiters in Table S3), report less overall sexual activity as adolescents while having similar levels of sexual activity as adults (Table 3), and report more STDS compared to unrelated controls (Table 4). Therefore, we postulate that individuals with FA might have an inadequate cellular response (notably natural killer cells) compared to controls. Longitudinal immune studies are needed to explore this hypothesis further.

Regardless of vaccination status, we also found that individuals with FA had a higher oral HPV prevalence. Indeed, among those testing positive, 83% were still oral DNA positive for a type targeted in the 4-plex vaccine post vaccination, suggesting that either vaccination does not completely protect individuals from oral HPV types included in the vaccine or exposure to and/or infection with HPV occurs before vaccination. As post-vaccination HPV seropositivity for the four HPV types targeted in the 4-valent Gardasil vaccine was not significantly different between FA and unrelated controls (Table 6), exposure to HPV before vaccination is the more likely reason for the observed differences in oral HPV prevalence, and not inadequate humoral or vaccination response.

To gain a better understanding of natural exposure to HPV, we examined the serology of unvaccinated children ≤13 years old with FA. Of the 46 children, 65% were seropositive to at least one of the nine HPV types targeted in the 9-valent Gardasil vaccine. Of the 19 total unvaccinated, unrelated controls ≤13 years old (58%) were also seropositive (Table S3). These data suggest that exposure to HPV is similar between FA and unrelated controls in childhood. As the earliest recommended vaccination age range for children is 9 to 11 years old, studies evaluating an earlier vaccine schedule for individuals with FA may be warranted. While the general population is similarly exposed in childhood, infection leading to pre-cancer and cancer is thought to be associated with HPV obtained via sexual contact [35]. For individuals with FA, earlier exposure to HPV through non-sexual routes might be equally concerning.

Considering this possibility, we observed that individuals with FA were also more likely to report being born prematurely and at lower birth weights (Table 3). Of those with FA, 13.6% who tested positive for oral HPV indicated that they were preterm at birth. Looking into the literature, one study of amniotic fluid obtained by amniocentesis and from rupture of membranes prior to cesarean delivery reported presence of HPV suggesting the possibility of prenatal infection in those without FA [36]; such findings have not, however, been confirmed by others [37]. HPV transmission is also said to occur when HPV on skin surfaces or fomites contacts microscopic injuries on the skin surface [38,39]. HPV shared via utensils can likewise enter the body through cuts or small tears inside of the mouth [40]. In our study, we observed individuals with FA to be more likely to report having had mouth sores over their lifetimes regardless of transplant status (Table 3). Those with FA also reported having less than ideal daily hygiene practices despite visiting the dentist more often and having more frequent dental cleanings/exams. It is impossible to know if the presence of mouth sores is a result of an inadequate mucosal barrier, is a result of mouth pain (or fear thereof), or possibly a result of differences in hygiene practices. However, having mouth sores could leave individuals with FA vulnerable to oral HPV infection.

To further complicate matters, individuals with FA are likely to require a hematopoietic stem cell transplant in childhood (average age 11 years old) [41]. Studies have detected HPV on reusable hospital equipment, such as ultrasound probes. This has raised concerns because some commonly used hospital disinfectants are ineffective against HPV (7,8). These studies indicate possible sources of HPV exposure during and after transplant. Those with FA are typically revaccinated for HPV after transplant, although there are differences in the time of revaccination based on clinical protocols. For our current study, the median time of sampling/testing since revaccination post-transplant was three years, a time-period that would allow for ample re-exposure to HPV. GMTs were generally lower in the vaccinated, transplanted FA group; but the differences compared to the vaccinated, non-transplanted group were statistically different in only three of the nine types (Table 7). These findings put forward the possibility that immune responses do not completely recover post-transplant thus opening the door for reinfection.

While immune factors are likely to be a critical factor, they alone do not explain the observed increase in HPV prevalence in FA. Indeed, tissue-specific mechanisms might also contribute. We have previously shown that increased accumulation of the HPV E7 protein, and/or viral replication in HPV16 and HPV31 positive 3D epidermal organoid models without intact FA DNA repair machinery results in greater HPV proliferation and/or viral load [9]. Other studies suggest that it is possible that loss of the FA DNA repair machinery expands the basal stem and progenitor compartment in human mucosa [21], a scenario that could also stimulate oral HPV prevalence and possibly enhance susceptibility to other viral infections, STDs and cancer development in FA. Others have shown that microabrasions in the surface epithelium of skin permit the entry of HPV into the cell where it targets the basal layer of the stratified squamous epithelium [39]. Indeed, our recent report demonstrates that the structure and function of the skin barrier of individuals with FA is disrupted [42]. A combination of differences in skin barrier, immune response and an inadequate DNA repair system, therefore, could make those with FA more vulnerable to HPV.

As with any observational study, there were a number of limitations. First, the age ranges of parent and sibling groups differed from those with FA. Not surprising, parents were much older and more likely to have had sex, and siblings were on average younger and less likely to have had sex. For this reason, we added an unrelated control group. Further, persistence of oral HPV did not significantly differ between individuals with FA and either the family or unrelated control groups (Table 2). This is not unexpected given our recruitment approach. While sample and data collection at both FARF events and hospital visits allowed us to expand our study’s sample size of individuals with FA and their family members, our partners did not allow us to collect and/or follow unrelated controls at these events. Therefore, most unrelated controls provided samples at our hospital at a single time point. Given FA is a rare disease and participants traveled from 15 countries and 34 states across the USA, family members were not always present to provide samples for testing. Additionally noteworthy, during the eight years of the study, at least 46 participants of 211 (21.7%) with FA died. This resulted in unequal follow-up samples per participant. Differences in health care providers’ willingness to share clinical files also forced us to rely on self-reported vaccine status and cancer histories. For this reason, it was not surprising that ever having cancer and HNSCC specifically was not significantly associated with oral HPV detection. Still, oral HPV was marginally positively associated with gynecological cancers in females with FA (*p* = 0.08). Future longitudinal studies are needed to further assess the risk of cancer in FA compared to family and/or unrelated controls.

## 5. Conclusions

Taken together, while the underlying molecular and cellular pathways have yet to be fully elucidated, we conclude that individuals with FA represent a population uniquely susceptible to HPV and perhaps other DNA tumor viruses, and that relevant mechanisms are independent of conventional risk factors. As individuals with FA are at considerably greater risk of HNSCC, identifying factors that augment risk of oral HPV DNA positivity could ultimately lead to viable preventive measures or interventions for those with FA and others with genome instability syndromes. Further mechanistic, observational and vaccination studies are needed to explore these possibilities.

## Figures and Tables

**Table 1:** Study Population Characteristics.

Characteristic	FA *n* = 212	Siblings *n* = 98	^a^*p*-Value	Parents *n* = 233	^b^*p*-Value	Unrelated *n* = 111	^c^*p*-Value	^d^ All Control *p*-Value
Age (years)			**<0.001**		**<0.001**		**0.048**	**<0.001**
≤11	93 (44%)	59 (60%)		0 (0%)		32 (29%)		
12–5	32 (15%)	17 (17%)		0 (0%)		25 (23%)		
16–20	25 (12%)	14 (14%)		0 (0%)		13 (12%)		
≥21	62 (29%)	8 (8%)		233 (100%)		41 (37%)		
Gender			0.87		0.26		0.61	0.48
Male	93 (44%)	42 (43%)		90 (39%)		52 (47%)		
Female	119 (56%)	56 (57%)		143 (61%)		59 (53%)		
Race			0.37		0.19		0.32	0.42
Black	10 (5%)	5 (5%)		10 (4%)		2 (2%)		
White	178 (84%)	86 (89%)		206 (89%)		99 (89%)		
Other	24 (11%)	6 (5%)		15 (6%)		10 (9%)		
Income			**0.016**		0.13		0.64	0.06
<40K	51 (26%)	9 (11%)		39 (19%)		25 (24%)		
40–90K	67 (35%)	30 (37%)		71 (34%)		42 (40%)		
>90K	76 (39%)	42 (52%)		98 (47%)		38 (36%)		
Insurance			**0.048**		**<0.001**		**<0.001**	**<0.001**
Private and public	26 (13%)	2 (2%)		7 (3%)		4 (4%)		
Private	115 (56%)	55 (68%)		145 (71%)		88 (81%)		
Public	55 (27%)	21 (26%)		38 (19%)		15 (14%)		
Self-pay	11 (5%)	3 (4%)		13 (6%)		2 (2%)		
^e^ Participant Education			0.32		**0.020**		0.07	**0.013**
<College	20 (27%)	3 (25%)		31 (14%)		11 (26%)		
College	43 (58%)	9 (75%)		131 (60%)		18 (42%)		
Post graduate	11 (15%)	0 (0%)		55 (25%)		14 (33%)		
^f,g^ Mother Education			0.34		-		**0.006**	**0.017**
<College	20 (16%)	7 (9%)		-		6 (10%)		
College	80 (64%)	50 (66%)		-		28 (47%)		
Post graduate	25 (20%)	19 (25%)		-		25 (42%)		
^f,g^ Father Education			0.21		-		0.61	0.40
<College	27 (23%)	9 (13%)		-		12 (21%)		
College	68 (57%)	45 (63%)		-		29 (52%)		
Post graduate	24 (20%)	18 (25%)		-		15 (27%)		

Note: Data are shown as frequency (%) and compared using chi-square tests. a: comparing Fanconi anemia (FA) with siblings; b: comparing FA with parents; c: comparing FA with unrelated controls; d: comparing FA to all control groups; e: for participants 18 years or older; f: for participants younger than 18 years old; g: parents were excluded from the comparison. Information taken from 1st non-missing time point. Bold indicates significant differences at the 0.05 level.

**Table 2:** Oral HPV DNA Positivity.

	FA (*n* = 201)	Siblings (*n* = 88)	^a^*p*-Value	Parents (*n* = 215)	^b^*p*-Value	Unrelated (*n* = 107)	^c^*p*-Value
Any Oral HPV Positive			**0.010**		0.07		**<0.001**
Yes	41 (20%)	7 (8%)		29 (13%)		6 (6%)	
No	160 (80%)	81 (92%)		186 (87%)		101 (94%)	
^d^ High Risk HPV			0.56		1.00		1.00
High Risk Positive	37 (90%)	6 (86%)		26 (90%)		6 (100%)	
Low Risk Positive	4 (10%)	1 (14%)		3 (10%)		0 (0%)	
^d^ HPV16			1.00		**0.045**		0.33
HPV16 Positive	31 (76%)	6 (86%)		15 (52%)		3 (50%)	
Other Type Positive	10 (24%)	1 (14%)		14 (48%)		3 (50%)	
^d^ HPV 6, 11, 16, 18 Positive			0.58		0.18		0.13
Yes	33 (80%)	7 (100%)		19 (66%)		3 (50%)	
No	8 (20%)	0 (0%)		10 (34%)		3 (50%)	
^e^ Multiple HPV Types			0.17		**0.045**		0.65
Yes	13 (32%)	0 (0%)		3 (10%)		1 (17%)	
No	28 (68%)	7 (100%)		26 (90%)		5 (83%)	
^f^ Incident HPV Positive			0.20		**0.048**		0.59
Yes	21 (18%)	3 (9%)		9 (9%)		0 (0%)	
No	93 (82%)	32 (91%)		96 (91%)		6 (100%)	
^g^ Type-specific Persistence			1.00		1.00		1.00
Yes	3 (2%)	0 (0%)		2 (2%)		0 (0%)	
No	126 (98%)	37 (100%)		112 (98%)		6 (100%)	
^h^ Consecutive Positives			0.59		1.00		1.00
Yes	5 (4%)	0 (0%)		4 (4%)		0 (0%)	
No	124 (96%)	37 (100%)		110 (96%)		6 (100%)	

Note: Data are shown as frequency (%) and compared using Fisher’s exact tests. a: comparing FA with siblings; b: comparing FA with parents; c: comparing FA with unrelated controls; d: subjects with no infection were excluded; e: participants tested oral HPV DNA positive (via Roche Linear Array) for multiple types within the same sample; f: subjects with oral HPV positive tests at first sampling as well as those without any oral HPV test results or those test results that were beta globin negative were removed from the analysis; g: examined in subjects who had more than one oral HPV test for the same type at multiple consecutive visits with no more than 1.5 years between visits. h: examined in subjects who had more than one oral HPV test at multiple consecutive visits. Bold indicates significant differences at the 0.05 level.

**Table 3:** Early and Later Life Exposures and Behaviors.

	FA (*n* = 212)	Siblings (*n* = 98)	^a^*p*-Value	Parents (*n* = 233)	^b^*p*-Value	Unrelated (*n* = 111)	^c^*p*-Value	^d^ All Control *p*-Value
Early Life Factors								
Delivery			0.06		**<0.001**		0.45	**<0.001**
Vaginal	144 (71%)	52 (59%)		191 (90%)		57 (76%)		
C-section	60 (29%)	36 (41%)		21 (10%)		18 (24%)		
Gestation			**0.033**		**<0.001**		**<0.001**	**<0.001**
Pre-term	56 (28%)	13 (15%)		14 (7%)		9 (10%)		
Full term	145 (72%)	71 (85%)		189 (93%)		82 (90%)		
Birthweight			**<0.001**		**<0.001**		**<0.001**	**<0.001**
<5.5 lbs	49 (35%)	2 (4%)		12 (9%)		6 (10%)		
≥5.5 lbs	91 (65%)	51 (96%)		119 (91%)		56 (90%)		
Breastfed			0.21		**<0.001**		0.53	**<0.001**
No	50 (27%)	15 (20%)		92 (52%)		17 (23%)		
Yes	133 (73%)	61 (80%)		86 (48%)		56 (77%)		
Hygiene Practices/Oral/Dental								
Brushing			0.49		**0.008**		0.06	**0.005**
Never	3 (2%)	2 (2%)		0 (0%)		0 (0%)		
<1X week	2 (1%)	1 (1%)		0 (0%)		0 (0%)		
At least 1X/week	13 (7%)	7 (8%)		6 (3%)		2 (3%)		
1-2 times daily	177 (91%)	74 (88%)		198 (97%)		73 (97%)		
Flossing			0.54		**<0.001**		**0.001**	**<0.001**
Never	73 (38%)	30 (36%)		16 (8%)		11 (15%)		
<1X/week	46 (24%)	17 (20%)		45 (22%)		21 (28%)		
At least 1X/week	46 (24%)	23 (27%)		92 (45%)		27 (36%)		
1-2 times daily	29 (15%)	14 (17%)		50 (25%)		15 (20%)		
Mouth sores			**<0.001**		**<0.001**		**<0.001**	**<0.001**
No	138 (70%)	78 (94%)		189 (91%)		71 (97%)		
Yes	58 (30%)	5 (6%)		18 (9%)		2 (3%)		
Dental Visits			0.57		**0.009**		0.63	**0.012**
<1 per year	35 (18%)	10 (12%)		42 (21%)		12 (16%)		
1 per year	29 (15%)	18 (21%)		48 (24%)		5 (7%)		
2 per year	104 (54%)	53 (63%)		106 (52%)		54 (72%)		
3 per year	24 (13%)	3 (4%)		8 (4%)		4 (5%)		
HPV/Oral Cancer Risk and Protective Factors								
Alcohol Use			0.25		**<0.001**		0.26	**<0.001**
No	179 (86%)	85 (91%)		135 (61%)		62 (81%)		
Yes	28 (14%)	8 (9%)		88 (39%)		15 (19%)		
Current Smoker			0.47		**<0.001**		0.72	**<0.001**
No	200 (98%)	89 (96%)		195 (88%)		95 (97%)		
Yes	5 (2%)	4 (4%)		27 (12%)		3 (3%)		
Ever Smoker			0.43		**<0.001**		0.69	**<0.001**
No	148 (84%)	63 (89%)		100 (56%)		59 (87%)		
Yes	28 (16%)	8 (11%)		78 (44%)		9 (13%)		
2nd Hand Smoke			0.25		0.44		0.46	0.29
No	120 (58%)	59 (65%)		117 (54%)		51 (53%)		
Yes	88 (42%)	32 (35%)		100 (46%)		45 (47%)		
^e^ Sexual History			**0.021**		**<0.001**		**0.042**	**<0.001**
No	139 (66%)	75 (80%)		0 (0%)		42 (53%)		
Yes	71 (34%)	19 (20%)		225 (100%)		38 (48%)		
^e^ HPV Vaccination			**<0.001**				0.13	**0.002**
No	116 (55%)	68 (76%)		219 (99%)		48 (66%)		
Yes	94 (45%)	21 (24%)		3 (1%)		25 (34%)		

Note: Data are shown as frequency (%) and compared using either Fisher’s exact tests (Early life factors, HPV oral cancer risk and protective factors variables) or Kruskal–Wallis tests (Hygiene Practices variables). a: comparing FA with siblings; b: comparing FA with parents; c: comparing FA with unrelated controls; d: FA compared to all control groups e: parents were excluded in the test. Bold indicates significant differences at the 0.05 level.

**Table 4:** Sexually Transmitted Disease History Among Sexually Active Participants.

	FA (*n* = 71)	Siblings (*n* = 19)	^a^*p*-Value	Parents (*n* = 224)	^b^*p*-Value	Unrelated (*n* = 37)	^c^*p*-Value	^d^ All Control *p*-Value
Ever had STD’s			0.14		**0.003**		**0.05**	**0.012**
No	46 (69%)	15 (88%)		171 (86%)		28 (88%)		
Yes	21 (31%)	2 (12%)		27 (14%)		4 (13%)		
Chlamydia			0.20		**0.004**		0.33	**0.020**
No	57 (85%)	17 (100%)		190 (96%)		30 (94%)		
Yes	10 (15%)	0 (0%)		8 (4%)		2 (6%)		
Genital HPV			0.06		**<0.001**		0.13	**0.001**
No	54 (81%)	17 (100%)		190 (96%)		30 (94%)		
Yes	13 (19%)	0 (0%)		8 (4%)		2 (6%)		
Herpes			1.00		0.19		0.17	0.27
No	62 (93%)	16 (94%)		191 (96%)		32 (100%)		
Yes	5 (7%)	1 (6%)		7 (4%)		0 (0%)		
Gonorrhea			0.37		1.00		0.54	0.20
No	66 (99%)	16 (94%)		196 (99%)		31 (97%)		
Yes	1 (1%)	1 (6%)		2 (1%)		1 (3%)		
Genital warts			1.00		1.00		1.00	1.00
No	66 (99%)	17 (100%)		194 (98%)		32 (100%)		
Yes	1 (1%)	0 (0%)		4 (2%)		0 (0%)		

Note: Data are shown as frequency (%) and compared using Fisher’s exact tests. a: comparing FA with siblings; b: comparing FA with parents; c: comparing FA with unrelated controls; d: FA compared to all control groups; Syphilis, Trichomonas, and other Miscellaneous STDS had <5 total positive subjects across the groups and were removed from the table (all had *p* = 1.00). Bold indicates significant differences at the 0.05 level.

**Table 5:** Potential Risk Factors in Individuals with FA by Oral HPV DNA Status.

		HPV DNA+	HPV DNA−	Crude OR (95% CI)	
Sex	Female	16	96	ref	
	Male	25	64	**2.34**	**(1.16–4.73)**
Enrollment age	<12 years	9	52	ref	
	12+ years	32	108	1.71	(0.76-3.85)
Race	White	40	130	ref	
	Non-white	1	30	**0.11**	**(0.01–0.82)**
Educational level	<College	28	96	ref	
	≥College	8	49	0.56	(0.24–1.32)
Gestational age	Full term	33	108	ref	
	Pre-term	6	44	0.45	(0.17–1.14)
Birthweight	≥5.5 lbs.	25	65	ref	
	<5.5 lbs.	7	40	0.46	(0.18–1.15)
Delivery method	Vaginal	30	108	ref	
	C-section	11	45	0.88	(0.41–1.91)
Breastfed	No	7	41	ref	
	Yes	28	100	1.64	(0.66–4.05)
Alcohol use	No	33	137	ref	
	Yes	8	20	1.66	(0.67–4.10)
Ever smoker	No	29	116	ref	
	Yes	8	20	1.60	(0.64–4.00)
Second hand smoke exposure	No	26	88	ref	
	Yes	14	70	0.68	(0.33–1.39)
HPV vaccination	No	18	90	ref	
	Yes	23	68	1.69	(0.85–3.38)
Sex experience	No	27	103	ref	
	Yes	14	55	0.97	(0.47–2.00)
Ever vaginal sex	No	28	105	ref	
	Yes	13	52	0.94	(0.45–1.95)
Ever give oral sex	No	29	108	ref	
	Yes	12	48	0.93	0.44–1.98)
Ever give anal sex	No	35	148	ref	
	Yes	3	5	2.54	0.58–11.12)
STD history	No	11	34	ref	
	Yes	4	16	0.77	(0.21–2.81)
Genital HPV	No	12	40	ref	
	Yes	3	10	1.00	(0.24–4.23)
Common warts	No	25	99	ref	
	Yes	13	36	1.43	(0.66–3.09)
Mouth sores	No	29	105	ref	
	Yes	11	44	0.91	(0.42–1.97)
Personal cancer history	No	34	135	ref	
	Yes	7	23	1.21	(0.48–3.05)
Family history of cancer	No	14	74	ref	
	Yes	27	77	1.85	(0.90–3.81)
Age of diagnosis of FA	<10 years	33	117	ref	
	≥10 years	5	25	0.71	(0.25–2.00)
History of HSCT	No	21	77	ref	
	Yes	20	83	0.88	(0.44–1.76)
FA complementation group	A	21	60	ref	
	Other	15	47	0.91	(0.42–1.96)

Among the 41 HPV+ individuals with Fanconi anemia (FA), all had data on HPV vaccination, 1 was missing data on mouth sores, 6 subjects were missing breastfeeding data. STD—sexually transmitted disease; HSCT—hematopoietic stem cell transplant. All participants had oral HPV DNA test results (via Roche Linear Array). Bold indicates significant differences at the 0.05 level.

**Table 6:** Seropositivity in Participants with FA Compared to Unrelated Controls.

HPV Type	Unvaccinated			Vaccinated		
	FA	Unrelated	*p*-Value	FA	Unrelated	*p*-Value
HPV6 (AU/mL)	35 (66%)	17 (53%)	0.26	52 (88%)	19 (90%)	1.00
HPV11(AU/mL)	27 (51%)	15 (47%)	0.82	52 (88%)	19 (90%)	1.00
HPV16 (IU/mL)	29 (56%)	14 (44%)	0.37	53 (88%)	19 (90%)	1.00
HPV18 (IU/mL)	27 (52%)	11 (34%)	0.18	44 (73%)	19 (90%)	0.13
HPV31(AU/mL)	19 (49%)	13 (41%)	0.63	32 (70%)	18 (86%)	0.23
HPV33 (AU/mL)	15 (38%)	12 (38%)	1.00	27 (59%)	17 (81%)	0.10
HPV45 (AU/mL)	17 (44%)	12 (38%)	0.64	29 (63%)	17 (81%)	0.17
HPV52 (AU/mL)	14 (36%)	12 (38%)	1.00	25 (54%)	17 (81%)	0.06
HPV58 (AU/mL)	19 (49%)	12 (38%)	0.47	**28 (61%)**	**18 (86%)**	**0.05**

Note: Data shown as frequency (%) and compared using Fisher’s exact test. Vaccinated subjects: FA = 60, Unrelated = 21. Unvaccinated subjects: FA = 52, Unrelated = 32. One subject who got bivalent vaccine was considered unvaccinated to HPV6 and HPV11. One subject who was vaccinated to 9 types was excluded from the analysis of HPV31, 33, 45, 52 and 58. IU/mL—International units per milliliter; AU/mL—Arbitrary units per milliliter.

**Table 7:** HPV Serotiters by HPV Vaccine and History of HSCT in Participants with FA.

HPV Type	Unvaccinated			Vaccinated		
	HSCT (−) (*n* = 29)	HSCT (+) (*n* = 24)	*p*-Value	HSCT (−) (*n* = 23)	HSCT (+) (*n* = 37)	*p*-Value
HPV6 (AU/mL)	0.76 (0.38, 1.50)	0.39 (0.18, 0.83)	0.18	29.27 (8.18, 104.70)	13.90 (5.73, 33.71)	0.33
HPV11 (AU/mL)	0.42 (0.22, 0.79)	0.26 (0.13, 0.51)	0.29	41.96 (11.53, 152.70)	12.60 (5.26, 30.19)	0.12
HPV16 (IU/mL)	1.29 (0.64, 2.60)	0.84 (0.38, 1.87)	0.41	117.60 (38.26, 361.20)	44.97 (17.28, 117.00)	0.19
HPV18 (IU/mL)	1.07 (0.65, 1.77)	0.81 (0.36, 1.85)	0.56	**44.05 (15.56, 124.70)**	**9.33 (3.55, 24.52)**	**0.029**
HPV31 (AU/mL)	1.75 (0.98, 3.13)	0.79 (0.38, 1.65)	0.08	**9.55 (4.63, 19.71)**	**3.35 (1.49, 7.52)**	**0.05**
HPV33 (AU/mL)	1.78 (0.99, 3.22)	1.01 (0.57, 1.79)	0.15	5.79 (2.73, 12.28)	2.31 (1.20, 4.45)	0.06
HPV45 (AU/mL)	3.37 (1.65, 6.87)	1.57 (0.78, 3.15)	0.12	10.48 (4.96, 22.16)	4.50 (2.26, 8.96)	0.09
HPV52 (AU/mL)	1.00 (0.61, 1.63)	0.76 (0.43, 1.33)	0.45	3.32 (1.54, 7.13)	1.28 (0.67, 2.45)	0.06
HPV58 (AU/mL)	3.49 (1.73, 7.03)	1.50 (0.68, 3.33)	0.10	**9.29 (4.16, 20.75)**	**2.68 (1.43, 5.02)**	**0.015**

Note: Data are shown as geometric mean (95%CI) and compared using t-tests. ELISA, titers based on either a 4-plex or 9-plex HPV VLP IgG enzyme-linked immunosorbent assay. Titers under the detection limits were imputed as half of the detection limits. One subject received a bivalent vaccine (Cervarix HPV16 & 18) and therefore was considered unvaccinated for HPV6 and HPV11. All but one remaining subject received Gardasil 4 vaccine. The single subject receiving Gardasil 9 vaccine was excluded from the analysis of HPV31, 33, 45, 52 and 58 (all below the table’s horizontal midline). HSCT—hematopoietic stem cell transplant. IU/mL—International units per milliliter; AU/mL—Arbitrary units per milliliter. Bold indicates significant differences at the 0.05 level.

## Data Availability

All data and remaining samples will be transferred to the Fanconi Anemia Repository (PI: P. Mehta) at Cincinnati Children’s Hospital Medical Center at the close of this study.

## Supplementary Materials

The following are available online at https://www.mdpi.com/2072-6694/13/6/1368/s1, Figure S1: Odds of Oral HPV Detection, Table S1: Group Comparisons of Sexual Behavior, Table S2: HPV Serotiters of Bone Marrow Transplant Negative Participants with FA Compared to Unrelated Controls, Table S3: HPV Titers for Unvaccinated Children ≤13 Years Old Without Reported Sexual Experience.
