# Supplementary material for: Human Papillomavirus Oral- and Sero- Positivity in Fanconi Anemia
Source: Cancers (Basel). 2021 Mar 18;13(6):1368. doi: 10.3390/cancers13061368 (PMC8003090; doi:10.3390/cancers13061368)
Supplement: Supplementary file 1 [file cancers-13-01368-s001.pdf]

cancers

MDPI

Supplementary Materials: Human Papillomavirus Oral- and Sero- Positivity in Fanconi Anemia

Sharon L. Sauter, Xue Zhang, Lindsey Romick-Rosendale, Susanne I. Wells, Kasiani C. Myers, Marion G. Brusadelli, Charles B. Poff, Darron R. Brown, Gitika Panicker, Elizabeth R. Unger, Parinda A. Mehta, Jack Bleesing, Stella M. Davies and Melinda Butsch Kovacic

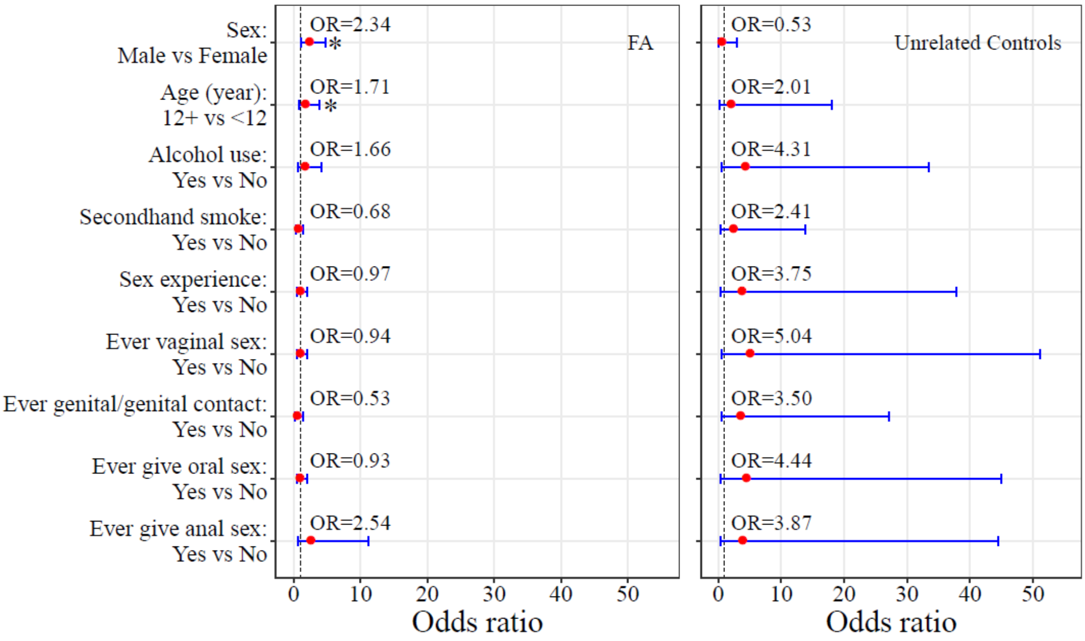

Figure S1. Odds of Oral HPV Detection. Included were 201 individuals with Fanconi anemia (FA; 41 oral HPV positive) compared to 107 unrelated controls (6 oral HPV positive).

Table S1. Group Comparisons of Sexual Behavior.

All participants	FA (N=212)	Siblings (N=98)	[a]p-value	Parents (N=232)	[b]p-value	Unrelated (N=110)	[c]p-value	[d]All Control p-value
[e]Kissing History			0.23		**<0.001**		0.13	0.07
No	123 (62%)	60 (70%)		8 (4%)		39 (52%)		
Yes	75 (38%)	26 (30%)		200 (96%)		36 (48%)		
Among Those With Prior Sexual Histories	FA (N=71)	Siblings (N=19)	[a]p-value	Parents (N=232)	[b]p-value	Unrelated (N=33)	[c]p-value	[d]All Control p-value
Ever had Vaginal Sex			0.11		**0.014**		0.65	**<0.001**
No	3 (4%)	3 (16%)		0 (0%)		2 (6%)		
Yes	68 (96%)	16 (84%)		218 (100%)		31 (94%)		
Genital to Genital Contact			0.74		0.75		0.63	0.80
No	19 (33%)	3 (23%)		58 (36%)		10 (38%)		
Yes	39 (67%)	10 (77%)		104 (64%)		16 (62%)		
Finger to Genital Contact			1.00		0.79		1.00	0.81
No	4 (7%)	0 (0%)		14 (9%)		1 (4%)		
Yes	55 (93%)	13 (100%)		149 (91%)		25 (96%)		
Ever Give Oral Sex			0.70		0.47		0.27	0.33
No	8 (11%)	3 (16%)		18 (8%)		1 (3%)		
Yes	62 (89%)	16 (84%)		197 (92%)		32 (97%)		
Ever Receive Oral Sex			0.19		0.32		0.27	0.33
No	8 (11%)	0 (0%)		16 (7%)		1 (3%)		
Yes	62 (89%)	19 (100%)		199 (93%)		32 (97%)		
Ever Give Anal Sex			0.68		1.00		0.34	0.49
No	55 (87%)	11 (85%)		153 (87%)		20 (77%)		
Yes	8 (13%)	2 (15%)		22 (13%)		6 (23%)		
Ever Receive Anal Sex			0.17		0.73		1.00	0.49
No	51 (76%)	15 (94%)		150 (79%)		20 (77%)		
Yes	16 (24%)	1 (6%)		41 (21%)		6 (23%)		

Note: Data are shown as frequency (%) and compared using Fisher's exact tests. a: comparing FA with siblings; b: comparing FA with parents; c: comparing FA with unrelated controls; d: comparing FA to all control groups; e: parents were excluded from the all group comparison. Those with missing responses were not included in individual analyses. Bold indicates significant differences at the 0.05 level.

Table S2. HPV Serotiters of Bone Marrow Transplant Negative Participants with FA Compared to Unrelated Controls.

HPV type	Unvaccinated			Vaccinated		
	HSCT (-) FA (n=29)	Unrelated (n=32)	p-value	HSCT (-) FA (n=23)	Unrelated (n=21)	p-value
HPV6 (AU/ml)	0.76 (0.38, 1.50)	0.40 (0.19, 0.86)	0.21	29.27 (8.18, 104.70)	43.16 (12.59, 147.90)	0.65
HPV11 (AU/ml)	0.42 (0.22, 0.79)	0.37 (0.17, 0.81)	0.78	41.96 (11.53, 152.70)	65.32 (18.17, 234.80)	0.61
HPV16 (IU/ml)	1.29 (0.64, 2.60)	0.84 (0.38, 1.88)	0.41	117.60 (38.26, 361.20)	120.8 (33.87, 431.10)	0.97
HPV18 (IU/ml)	1.07 (0.65, 1.77)	0.86 (0.42, 1.75)	0.60	44.05 (15.56, 124.70)	35.48 (11.68, 107.70)	0.77
HPV31 (AU/ml)	1.75 (0.98, 3.13)	1.27 (0.73, 2.22)	0.42	9.55 (4.63, 19.71)	12.22 (4.92, 30.37)	0.66
HPV33 (AU/ml)	1.78 (0.99, 3.22)	1.57 (0.92, 2.67)	0.74	5.79 (2.73, 12.28)	7.60 (3.20, 18.04)	0.62
HPV45 (AU/ml)	3.37 (1.65, 6.87)	2.11 (1.33, 3.33)	0.26	10.48 (4.96, 22.16)	12.69 (5.97, 26.98)	0.71
HPV52 (AU/ml)	1.00 (0.61, 1.63)	1.07 (0.63, 1.82)	0.84	3.32 (1.54, 7.13)	5.07 (2.14, 12.00)	0.45
HPV58 (AU/ml)	3.49 (1.73, 7.03)	2.17 (1.27, 3.71)	0.27	9.29 (4.16, 20.75)	12.08 (5.49, 26.56)	0.63

Note: Data are shown as geometric mean (95% CL) and compared using t-tests. ELISA, titers based on either a 4-plex or 9-plex HPV VLP IgG enzyme-linked immunosorbent assay Titers under detection limits were imputed as half of the detection limits. One subject who received Cervarix (bivalent) vaccine was considered unvaccinated to HPV6 and HPV11. One subjected who received the Gardasil 9 vaccine was excluded from the analysis on HPV31, 33, 45, 52 and 58. HSCT (-) indicates subjects who had not reported a history of a hematopoietic stem cell transplant. IU/ml – International units per milliliter; AU/ml – Arbitrary units per milliliter.

Table 3. HPV Titers for Unvaccinated Children ≤13 Years Old Without Reported Sexual Experience.

Group	Characteristics		M4/M9 ELISA (IU/ml)		M4/M9 ELISA (AU/ml)							Ever Oral HPV+
	Age (years)	HSCT	HPV16	HPV18	HPV6	HPV11	HPV31	HPV33	HPV45	HPV52	HPV58	
FA (n=30; 65% of those age <=13yrs)	2	No	1.8	2.3	0.5	0.5	5.3	4.3	8.2	2.4	9.6	HPV16
	5	No	1.0	1.3	0.4	0.5	2.4	2.7	3.6	1.8	3.9	Neg
	5	No	0.6	1.3	0.4	0.3	2.3	2.6	4.7	1.7	3.6	Neg
	5	No	0.4	0.8	0.3	0.3	1.16	1.49	1.38	Neg	1.56	HPV84
	6	No	7.6	3.4	3.3	3.4	3.2	3.4	4.8	1.5	4.9	Neg
	6	No	2.6	3.6	0.3	0.2	6.5	4.3	17.6	2.0	13.6	HPV16
	6	No	4.1	8.9	2.8	4.9	16.8	23.9	17.2	10.0	13.7	Neg
	6	No	7.4	2.2	6.0	6.4	3.2	1.7	3.4	0.8	3.5	Neg
	6	Yes	1.0	1.2	0.3	0.3	2.3	2.1	4.4	2.3	4.4	Neg
	6	Yes	1.4	2.2	0.6	0.5	3.8	3.8	8.8	2.3	7.1	Neg
	7	No	2.2	1.6	0.9	0.6	2.1	1.6	2.7	0.8	3.0	Neg
	7	Yes	0.57	0.51	1.25	1.18	N/A	N/A	N/A	N/A	N/A	HPV66
	8	Yes	4.8	2.2	2.0	1.6	2.1	2.1	3.2	1.3	4.0	Neg
	8	No	2.0	1.8	0.5	0.2	N/A	N/A	N/A	N/A	N/A	Neg
	8	No	1.11	1.38	0.38	0.46	N/A	N/A	N/A	N/A	N/A	Neg
	8	No	2.0	3.1	0.8	0.8	6.1	4.8	12.2	2.5	10.3	Neg
	8	No	0.7	1.2	0.4	0.5	2.2	2.6	2.5	1.2	2.7	Neg
	9	Yes	0.6	1.1	0.7	0.4	2.4	2.4	4.0	1.1	4.1	Neg
	9	No	4.6	4.7	0.8	0.6	9.6	7.3	20.7	4.6	22.7	Neg
	9	No	3.9	4.5	1.1	1.2	N/A	N/A	N/A	N/A	N/A	HPV16
	9	Yes	15.5	19.5	4.7	1.6	N/A	N/A	N/A	N/A	N/A	HPV6, 16
	9	No	57.5	20.6	9.5	10.6	8.0	8.4	15.1	4.6	10.9	Neg
	10	Yes	Neg	Neg	1.1	0.2	Neg	Neg	Neg	Neg	Neg	Neg
	10	Yes	1.6	1.8	0.8	1.2	N/A	N/A	N/A	N/A	N/A	Neg
	10	Yes	2.3	3.5	0.6	0.4	6.4	4.8	11.7	3.1	14.1	Neg
	11	Yes	0.4	1.0	0.3	0.3	2.0	2.0	3.4	1.1	3.3	Neg
	12	No	2.9	3.5	0.5	0.4	6.3	6.0	16.3	2.9	14.8	Neg
	13	Yes	0.9	1.6	0.4	0.4	2.5	3.4	4.6	2.0	4.9	Neg
	13	No	0.3	0.6	0.3	0.3	1.3	1.4	1.8	-	1.5	Neg
	13	No	21.5	4.4	7.7	34.0	N/A	N/A	N/A	N/A	N/A	HPV6, 16
Unrelated (n=11; 58% of those age <=13yrs)	4	N/A	2.6	3.5	0.4	0.4	6.7	4.7	8.9	2.9	16.2	Neg
	6	N/A	2.7	3.1	0.7	0.7	6.3	6.6	12.2	4.2	14.6	Neg
	6	N/A	1.1	0.8	0.3	0.4	2.0	2.6	4.1	1.4	4.3	Neg
	7	N/A	0.6	0.9	0.3	0.3	1.4	1.9	1.9	1.6	2.1	Neg
	9	N/A	1.1	1.0	0.4	0.4	2.6	2.9	3.9	2.0	5.0	Neg
	11	N/A	0.7	1.1	0.4	0.4	2.0	2.1	3.7	1.1	4.0	Neg
	11	N/A	0.7	1.3	0.5	0.5	2.3	2.8	3.1	1.4	2.7	Neg
	11	N/A	1.4	2.1	0.6	0.6	3.1	4.7	6.0	2.8	7.2	HPV16
	12	N/A	44.8	10.2	6.1	6.4	26.4	24.5	7.6	117.0	92.3	Neg
	12	N/A	1.4	2.0	0.5	0.5	3.4	4.6	6.5	2.3	6.9	Neg
	12	N/A	1.5	2.5	0.9	1.1	4.3	5.6	5.8	4.3	5.4	Neg

Note: Only unvaccinated children with at least one HPV positive serological result were included. Vaccination status was based on self-report, but verified by medical records where available. Titers based on either a 4-plex (N=7 children with FA) or 9-plex HPV VLP IgG enzyme-linked immunosorbent assay (ELISA). Most recent time points were used if more than one sample had been collected prioritizing before transplant samples over post-transplant samples. HSCT - hematopoietic stem cell transplant. N/A indicates not applicable. Neg – indicates a negative result. Self-reported sexual activity is a known limitation to the study.
